# N6-Methylandenosine-Related lncRNAs Predict Prognosis and Immunotherapy Response in Bladder Cancer

**DOI:** 10.3389/fonc.2021.710767

**Published:** 2021-08-11

**Authors:** Yuying Zhang, Baoyi Zhu, Minghui He, Yi Cai, Xiaoling Ying, Chonghe Jiang, Weidong Ji, Jianwen Zeng

**Affiliations:** ^1^Department of Urology, The Sixth Affiliated Hospital of Guangzhou Medical University (Qingyuan People’s Hospital), Qingyuan, China; ^2^Department of Liver Surgery, The First Affiliated Hospital of Sun Yat-sen University, Guangzhou, China; ^3^Department of Urology, Xiangya Hospital, Central South University, Changsha, China; ^4^Center for Translational Medicine, The First Affiliated Hospital, Sun Yat-sen University, Guangzhou, China

**Keywords:** bladder cancer, N6-methyladenosine, lncRNA, prognosis, tumor microenvironment, immune response

## Abstract

Both lncRNAs and the N6-methyladenosine (m6A) modification are key regulators of tumorigenesis and innate immunity. However, little is known about the m6A modification of lncRNAs and their clinical and immune relevance in bladder cancer. In this study, we identified m6A-related lncRNAs using Pearson correlation analysis in The Cancer Genome Atlas (TCGA) and the IMvigor210 datasets. Next, univariate Cox regression was performed using the TCGA dataset to filter prognostic m6A-related lncRNAs, which were further subjected to the least absolute shrinkage and selection operator (LASSO) Cox regression to establish a 12 m6A-related lncRNA prognostic score (m6A-LRS). The m6A-LRS was validated in the IMvigor210 dataset. In addition, high m6A-LRS tumors, characterized by decreased tumor mutation load and neoantigen load, showed poorer response to immunotherapy than those with low m6A-LRS in the IMvigor210 dataset. Further, we constructed an m6A-LRS-based nomogram that demonstrated a strong ability to predict overall survival in patients with bladder cancer. Moreover, enrichment analysis revealed that tumor-associated biological processes, oncogenic signaling, and tumor hallmarks were commonly associated with a high m6A-LRS. Gene set variation analysis also indicated that high m6A-LRS was associated with activation of canonical oncogenic signatures, such as the epithelial-to-mesenchymal transition, cell cycle regulators, and DNA replication, as well as activation of immunosuppressive signatures, such as the T-cell exhaustion and pan-fibroblast-TGF-β response signatures. Furthermore, we observed distinct tumor microenvironment cell infiltration characteristics between high- and low-risk tumors. High m6A-LRS tumors showed reduced infiltration of CD8+ T-cells and enhanced infiltration of macrophages and fibroblasts. Additionally, we established a competing endogenous RNA network based on the12 m6A-related lncRNAs. Finally, three lncRNAs (SNHG16, SBF2-AS1, and BDNF-AS) were selected for further validation. The qualitative PCR assay on 10 pairs of bladder cancer and adjacent normal control samples validated the differential expression, and methylated RNA immunoprecipitation (MeRIP) analysis demonstrated a robust m6A enrichment in T24 bladder cancer cells compared with normal uroepithelial cells (SVHUC-1). In conclusion, this study introduced an m6A-related lncRNA signature that identified a subgroup of patients with poor prognoses and suboptimal immune responses, thus providing novel approaches for treatment response prediction and patient stratification in bladder cancer.

## Introduction

Bladder cancer is the most common malignancy of the urinary system. Approximately 75% of bladder cancers are non-muscle-invasive tumors characterized by high frequency of recurrence, whereas the remaining 25% are muscle-invasive tumors characterized by poor prognosis (1). Despite the great improvement in antitumor treatment in the past three decades, the 5-year survival rate of muscle-invasive bladder cancer remains largely unchanged (2, 3). In recent years, immunotherapy with checkpoint inhibitors has revolutionized cancer treatment; however, the response rate remains unsatisfactory with only a small proportion of patients responding to this treatment (4, 5). Thus, the discovery of novel therapeutic targets and formulation of personalized treatment strategies for bladder cancer is urgently required.

N6-methyladenosine (m6A), the most abundant modification of mRNA and noncoding RNAs in eukaryotes, is vital for RNA splicing, stability, intracellular distribution, and translation (6, 7). The cellular m6A methylation is a highly dynamic status, which is mediated by a group of regulators, namely “writers” (m6A methylases), “erasers” (m6A demethylases), and “readers” (signal transducers). Recent studies have demonstrated that m6A modifications play important roles in regulating a variety of cellular processes, such as embryonic development (8), stem cell expansion (9), and tumorigenesis (10). For instance, methyltransferase like 3 (METTL3) has been suggested to be involved in the chemical carcinogenesis of bladder cancer *via* m^6^A methylation of CDCP1 (11). However, METTL14 has been reported to inhibit tumorigenesis through the m6A modification of Notch1, inhibiting its mRNA stability in bladder cancer (12). Recently, studies have also emerged revealing regulation of tumor microenvironment (TME), cell infiltration, immune activity, and the antitumor efficacy of immune checkpoint inhibitors through m6A modifications (13). Nevertheless, the relationship of m6A modification with cancer progression and immune response remains unclear in bladder cancer.

Long noncoding RNAs (lncRNAs) are important regulators of gene expression in cell growth, differentiation, proliferation, and survival (14, 15). The dysregulated expression of lncRNAs has been shown to be critical in the pathogenicity of malignant tumors, including bladder cancer (16–18). For instance, lncRNA SLC16A1-AS1 has been reported to promote metabolic reprogramming and invasiveness through its dual action as both a target and co-activator of E2F1 in bladder cancer (19). Likewise, lncRNA LNMAT1 was shown to promote bladder cancer lymphatic metastasis *via* upregulation of CCL2 and recruitment of macrophages into tumors (18). Recently, some studies have suggested an indispensable role of m6A modifications in the dysregulation of lncRNAs during tumorigenesis (20, 21). However, the role and the mechanisms underlying m6A modification in the dysregulation of lncRNAs in bladder cancer remain unknown.

In this study, we aim to investigate the clinical significance and potential regulation mechanisms underlying m6A modification of lncRNAs in bladder cancer. By employing The Cancer Genome Atlas (TCGA) dataset (n=402) and the immunotherapy cohort IMvigor210 (n=348) (22, 23), we identified m6A-related lncRNAs and constructed an m6A-related lncRNA risk score that exhibited robust prognostic ability with potential in identifying patients with a favorable response to immunotherapy. Additionally, an m6A-lncRNAs-based nomogram was constructed to predict the overall survival of patients with bladder cancer. We then explored the potential functions and pathways of m6A-related lncRNAs in relation to oncogenic signaling and immune regulation. A competing endogenous RNA (ceRNA) network was built to search for the potentially targeted miRNAs and mRNAs of the identified m6A-related lncRNAs. Finally, we selected three identified m6A-related lncRNAs for further validation with quantitative PCR and methylated RNA immunoprecipitation (MeRIP).

## Materials and Methods

### Clinical Samples

A total of ten pairs of bladder cancer and adjacent normal control samples were collected from patients in The Sixth Affiliated Hospital of Guangzhou Medical University from 2018 to 2020. This research was approved by the Medical Ethics Committee of The Sixth Affiliated Hospital of Guangzhou Medical University and informed consent was obtained from all patients.

### Datasets

The RNA-seq and clinicopathological data of TCGA and the GTEX RNA-seq data were downloaded from the USCS Xena website (https://xena.ucsc.edu/welcome-to-ucsc-xena). We combined the TCGA RNA-seq and GTEX RNA-seq data (n=9) for differential expression analysis between tumor and normal control samples. The IMvigor210 study evaluated the efficacy and safety of atezolizumab, a PD-L1-targeting antibody, in patients with platinum-treated locally advanced or metastatic urothelial carcinoma (22, 23). The RNA-seq profile and clinical data were obtained from http://research-pub.gene.com/IMvigor210CoreBiologies/, and the raw count data were transformed into transcripts per kilobase million (TPM) values for analysis. After excluding those without complete survival data, a total of 402 patients in the TCGA cohort and 348 patients in the IMvigor210 cohort were included in this study. The gene expression values were log2(TPM+1) transformed for subsequent analysis. The clinicopathological and molecular characteristics of the samples included in the current study are provided in **Supplementary Tables S1**, **S2**.

### Annotation of Long Noncoding RNAs

The lncRNA annotation file of GRCh38 was obtained from the GENCODE website (https://www.gencodegenes.org). A total of 14247 and 2322 lncRNAs were identified in the TCGA and IMvigor210 datasets, respectively. In the current study, we analyzed the 2322 shared lncRNAs identified in both datasets. The lncRNA types included lincRNAs, processed transcript, antisense, sense intronic, sense overlapping, 3’ overlapping ncRNAs, and macro lncRNAs.

### Identification of m6A-Related lncRNAs

Based on the literature, 23 well-recognized m6A regulators were identified, including eight writers (*METTL3, METTL14, METTL16, WTAP, VIRMA [KIA1429], RBM15, RBM15B*, and *ZC3H13*), two erasers (*FTO* and *ALKBH5*), and 11 readers (*YTHDC1, YTHDC2, YTHDF1, YTHDF2, YTHDF3, HNRNPA2B1, HNRNPC, IGF2BP1, IGF2BP2, IGF2BP3, RBMX, LRPPRC*, and *FRM1*) (24, 25). As shown in **Supplementary Figure S1**, aberrant dysregulations of the mRNA expression levels of these genes were observed in TCGA bladder cancer samples. Pearson correlation analysis was performed in each dataset to identify m6A-related lncRNAs which showed significant correlation (correlation coefficient > 0.3 and P-value < 0.001) with any one of the m6A-related regulators. A total of 581 and 196 lncRNAs were identified in the TCGA and IMvigor210 datasets, respectively. Of these, 143 shared lncRNAs were used for further analysis.

### Construction of m6A-Related Long Noncoding RNA Prognostic Score

Univariate Cox regression analysis was performed to filter prognostic lncRNAs in the TCGA dataset. In the current study, lncRNAs were divided into high- and low-expression subgroups using the optimal cut-off value determined by the “surv_cutpoint” function in the “survminer” R package. Next, significant prognostic lncRNAs with P-value <0.01 were subjected to the least absolute shrinkage and selection operator (LASSO) Cox regression with the penalty parameter estimated by 10-fold cross-validation using the “glmnet” R package. A total of 12 lncRNAs were identified using the minimum lambda value, and the m6A-related lncRNA risk score (m6A-LRS) was calculated using the following formula (25):

$$\text{m}6\text{A}-\text{LRS}=\Sigma_{i=1}^{n}Coefi*xi,$$

where *Coefi* indicated the coefficient, and *xi* is the log2(TPM+1) value of each m6A-related lncRNA.

### Molecular Classifier, Tumor Neoantigen Burden, and Tumor Mutation Burden

Based on mRNA expression data, bladder cancer was characterized into five molecular subtypes (luminal papillary, luminal, luminal infiltrated, basal/squamous, and neuronal) in the TCGA dataset (26). HLA binding predictions were employed to rank peptides, and the tumor neoantigen burden (TNB) was defined as the total number of predicted peptide/allele binders with a rank percentile score less than or equal to the weak binder threshold (2%) (26). In the current study, we extracted molecular subtypes and TNB data from the supplementary data of a previous study (26). Additionally, we obtained the TCGA mutation dataset from UCSC Xena and calculated the tumor mutation burden (TMB) using the number of non-synonymous somatic mutations (single nucleotide variants, frameshift, and nonsense mutation) per megabase (Mb) in coding regions. In the IMvigor210 dataset, the tumor samples were classified into four subtypes (I, II, III, IV) according to TCGA taxonomy, and five subtypes (basal/squamous cell carcinoma [SCC]-like, genomically unstable, infiltrated, urothelial-like A, and urothelial-like B) according to Lund taxonomy. As for TMB and TNB, we directly used the values provided in the supplementary data of the study (23), in which TMB was estimated using the FoundationOne^®^ TMB panel. The molecular subtypes, TMB and TNB data were extracted from the following website: http://research-pub.gene.com/IMvigor210CoreBiologies/.

### Functional Enrichment and Gene Set Enrichment Analysis in TCGA Cohort

Corresponding m6A-LRS was calculated for all patients. The “limma” R package was applied to determine the differentially-expressed genes (DEGs) between the top 25% and bottom 25% subgroups according to the risk score. Significant DEGs (log_2_|fold change|>1 and P-value <0.001) were subjected to Gene Ontology (GO) and Kyoto Encyclopedia of Genes and Genomes (KEGG) pathway enrichment analyses using the R package “clusterProfiler”. In addition, gene set enrichment analysis (GSEA) was performed to identify KEGG pathways and tumor hallmarks associated with m6A-LRS. The KEGG gene sets (“c2.cp.kegg.v7.2.entrez.gmt”) and hallmark gene sets (“h.all.v7.2.entrez.gmt”) were obtained from the Broad Institute database, and the R package “gseaplot2” was used to draw the GSEA plot.

### Estimation of Tumor-Microenvironment Cell Infiltration

The CIBERSORT algorithm (https://cibersort.stanford.edu/) was used to estimate the relative abundances of 22 distinct immune cell types based on gene expression in tumor tissues (27). We also applied the microenvironment cell population-counter (MCP-counter) method to quantify the absolute abundance of eight immune and two stromal cell populations within tumor tissues from the RNA-seq data using the R package “MCPcounter” (28). Significant different cell types between high and low m6A-LRS tumors were further subjected to univariate Cox regression analysis to determine their association with overall survival using the best cut-off value for grouping.

### Gene Set Variation Analysis

We performed gene set variation analysis (GSVA) using “GSVA” R packages to estimate the variation in biological process activities in the studied samples based on the RNA-seq data in TCGA. The gene sets used in this study included the “activated stromal genes” (29); “Wnt-TGF-β signature”; “T-cell TGF-β response signature (T-TRBS)” (30), “T-cell exhaustion signature” (31); and the gene sets constructed by Mariathasan et al. in the IMvigor210 study (23), including “CD8 T-effector signature”; “immune-checkpoint”; “antigen processing machinery”; “epithelial-to-mesenchymal transition (EMT) markers including “EMT1”, “ EMT2”, and “EMT3”; “pan-fibroblast TGF-β response signature (Pan-F- TBRS)”; “Wnt targets”; “DNA damage repair”; “mismatch repair”; “nucleotide excision repair”; “DNA replication”; “cell cycle”; “cell-cycle regulator”; “histone”; and “homologous recombination”. Study samples were divided into high- and low-risk subgroups according to the median value of m6A-LRS and comparisons were performed on the signature scores of the gene sets between groups.

### Construction of Competing Endogenous RNA Network

The 12 lncRNAs were used as seeds for the enrichment of lncRNA-miRNA interactions according to the analysis of miRanda (http://www.mirorna.org/). The miRNA-mRNA interactions were searched in the miRTarBase database (http://mirtarbase.cuhk.edu.hk/). Then, cytoscape (http://www.cytoscape.org/) was applied to build the lncRNA-miRNA-mRNA interaction ceRNA network. The mRNA gene list was subjected to the Metascape online tool (https://metascape.org/) for functional enrichment analysis.

### SRAMP Prediction of m6A Modification Site on lncRNAs

The SRAMP (Sequence-based RNA Adenosine Methylation site Predictor) is an online tool that predicts m6A modification site based on the RNA sequences of interests using machine learning framework (http://www.cuilab.cn/sramp). SRAMP was proved to have promising performance (32). In our study, SRAMP was used to predict m6A methylation site on the 12 m6A-related lncRNAs.

### Cell Cultures

Human uroepithelial cells (SV-HUC-1) and human bladder cancer cells T24 were purchased from the Institute of Cell Biology, Chinese Academy of Sciences (Shanghai, China). SV-HUC-1 cells were cultured in Ham’s F-12K (Kaighn’s) medium (Gibco, USA) and T24 cells were cultured in RPMI1640 medium (Gibco, USA) in a water-saturated atmosphere with 5% CO2 at 37°C. All medium was supplemented with 10% foetal bovine serum (Gibco, USA).

### RNA Isolation and Quantitative Real-Time PCR

Total RNA was extracted using TRIzol (Invitrogen) according to the manufacturer’s instructions. PrimeScript™ RT Reagent Kit with gDNA Eraser (Takara) was used for cDNA synthesis. Quantitative real-time PCR (qPCR) using Fast SYBR Green PCR Master Mix (Applied Biosystems) was performed on a Step-One Fast Real-time PCR System (Applied Biosystems). The 2-^△△CT^ method was used for data analyses. The primers were designed surrounding the SRAMP-predicted m6A-modification site and sequences are shown as bellow:

BDNF-AS-F: 5’-ACAGCAGAACAGATGGTCCG-3’;
BDNF-AS-R: 5’-ATTCATGCTGAGGGCCTCTG-3’;
SBF2-AS1-F: 5’- AATGTAGTCTGAACTCACACTTTTCAC-3’;
SBF2-AS1-R: 5’-TAATGACTAATGTGTTTATGGACAGAG-3’;
SNHG16-F: 5’- GGAAAACCTTTTGGCAGTTAGTGA-3’;
SNHG16-R: 5’- CTGGGGTTAAGCATTACTCTCT-3’.

### Methylated RNA Immunoprecipitation

The MeRIP assay was performed as reported in our previous study (33). Briefly, anti-m6A primary antibody (Synaptic Systems) was incubated with Pierce™ Protein A/G Magnetic Beads (Thermo Scientific) at 4°C for 3 h. Then, RNA was fragmented with an RNA fragmentation kit (Ambion) and then incubated with the mixture of Protein A/G and m6A-antibody at 4°C overnight. The immunoprecipitated RNA was washed five times and eluted from beads with m6A nucleotide solution (Sigma-Aldrich) and then purified for RT-qPCR assays.

### Statistical Analyses and Nomogram Construction

The R package (version 4.0.2) was used for statistical analyses in this study. The Student’s *t*-test, Wilcoxon test, Kruskal-Wallis test, and chi-square test were used for between-group comparisons as appropriate. Kaplan–Meier curve with log-rank test was used to compare the overall survival and progression-free survival between the high- and low-risk subgroups determined by the median value of m6A-LRS. To validate the robustness of m6A-LRS in predicting survival, we also performed a sensitivity analysis by 100 times random splits of TCGA samples into two subgroups (ratio 2:1) for survival analysis. In addition, multivariate Cox regression analyses were used to evaluate the independent prognostic value of m6A-LRS on overall survival. Then, significant variables in the multivariate Cox regression were subjected to nomogram construction with the “rms” R package in the TCGA dataset. The calibration plots (1, 3, and 5 years) and C-index were used to evaluate the nomogram’s prognostic accuracy.

## Results

### Identification of m6A-Related lncRNAs

**Figure 1:**
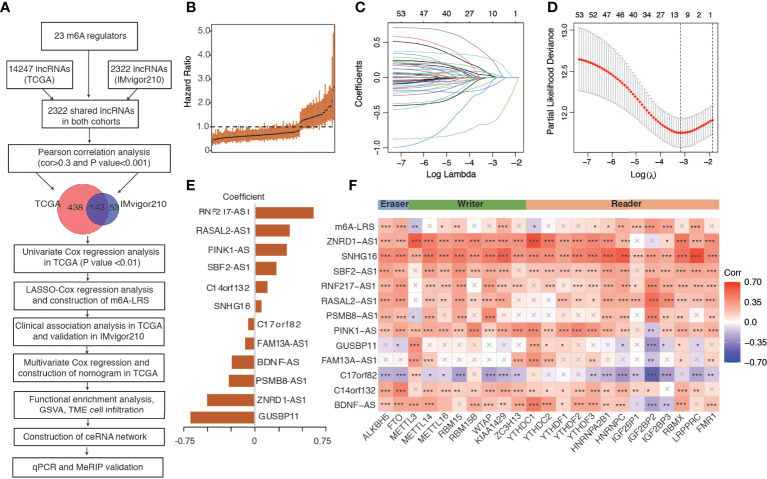
Study flowchart and construction of the m6A-related lncRNA risk score. **(A)** Study flowchart. **(B)** The hazard ratio of the 143 m6A-related lncRNAs for overall survival in the TCGA cohort. Error bars indicate 95% confidence intervals. **(C, D)** Least absolute shrinkage and selection operator (LASSO) regression model. **(E)** Coefficients of the LASSO-Cox model with the minimum lambda criteria. **(F)** Heatmap of the correlations between m6A regulators, 12 prognostic m6A-related lncRNAs, and the corresponding risk score. An “X” indicates a non-significant correlation (P > 0.05), *P < 0.05, **P < 0.01, ***P < 0.001. m6A-LRS: m6A-related lncRNA risk score.

Using the “gencode.v22.annotation”, we identified 14247 lncRNAs in the TCGA dataset and 2322 lncRNAs in the IMvigor210 dataset. We then used the shared 2322 lncRNAs for subsequent analysis. Through Pearson correlation analysis, we identified 581 and 196 m6A-related lncRNAs (defined as |Pearson R| > 0.3 and P < 0.001) in the TCGA and IMvigor210 datasets, respectively. We performed univariate Cox regression analysis on the 143 shared m6A-related lncRNAs to filter overall survival-related prognostic lncRNAs in the TCGA dataset, and then utilized the 53 significant lncRNAs with a p-value <0.01 for further analysis. The workflow process is outlined in **Figure 1A**, and the hazard ratio (95% confidence interval) of each prognostic m6A-related lncRNA in the TCGA dataset is shown in **Figure 1B**.

### Construction of m6A-Related lncRNA Risk Score

We performed LASSO-Cox regression analysis on the identified significant m6A-related prognostic lncRNAs to construct a risk score for the prediction of overall survival. As shown in **Figures 1C, D**, using the minimum lambda criteria, 12 lncRNAs were selected for the construction of our model. The coefficient of each lncRNA is shown in **Figure 1E**, while the survival plots are shown i**n** **Supplementary Figure S2**. Subsequently, we calculated the corresponding m6A-related lncRNA risk score (m6A-LRS) for each patient. The correlations between m6A-LRS, lncRNAs, and m6A regulators are shown in **Figure 1F**.

### Clinicopathological Characteristics and Molecular Subtypes Associated With m6A-LRS in TCGA

**Figure 2:**
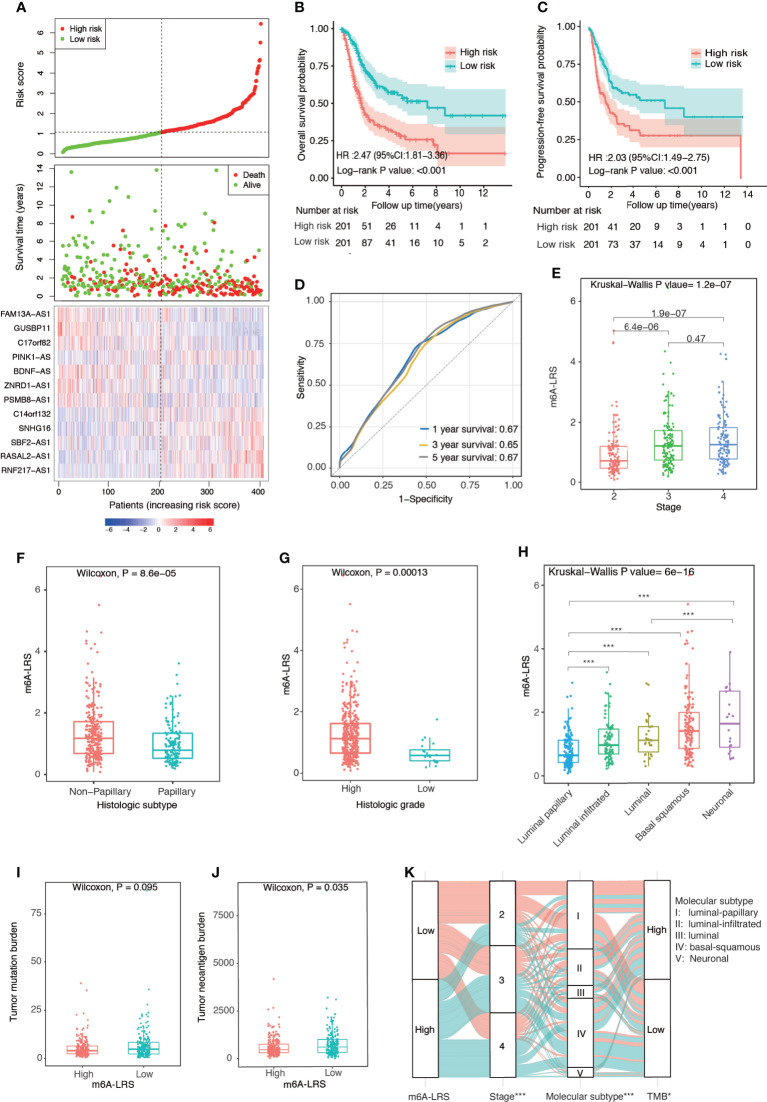
Clinical relevance of the m6A-related lncRNA risk score in the TCGA dataset. **(A)** Distributions of the m6A-related lncRNA risk score (m6A-LRS), survival status, and the expression levels of 12 prognostic m6A-related lncRNAs in patients with bladder cancer in the TCGA cohort. **(B, C)** Kaplan–Meier curves reveal worse overall survival and progression-free survival in the high-risk than the low-risk subgroup. **(D)** Receiver operating characteristic (ROC) curves of m6A-LRS for predicting the 1/3/5-year survival in the TCGA dataset. **(E–H)** Comparison of m6A-LRS-based subgroups with regard to clinical stage **(E)**, histological subtype **(F)**, histological grade **(G)**, and molecular subtype **(H)**. **(I, J)** Comparison of tumor mutation burden (TMB) and tumor neoantigen burden (TNB) between the high- and low-risk subgroups. **(K)** Alluvial diagram showing the changes in m6A-LRS, clinical stage, molecular subtypes, and TMB. TMB was stratified into high- and low-TMB group according to the median value. *P < 0.05, ***P < 0.001 for chi-square test between m6A-LRS subgroup and the corresponding variables.

Patients in the TCGA cohort were divided into high- and low-risk subgroups based on the median value of m6A-LRS. Patients with higher m6A-LRS had poorer overall survival and progression-free survival (**Figures 2A–C**). The ROC curves of m6A-LRS for the prediction of 1, 3, and 5-year overall survival in the TCGA cohort are shown in **Figure 2D**. Patients with advanced pathological stage, non-papillary subtype, or high histologic grade were more likely to have a higher score (**Figures 2E–G** and **Supplementary Table S1**). In addition, among the five molecular subtypes, the neuronal subtype had the highest value of m6A-LRS, followed by the basal squamous subtype, while the luminal-papillary subtype had the lowest score (**Figures 2H**). Moreover, TNB and TMB were lower in m6A-LRS-high tumors (**Figures 2I–K**). Interestingly, low TMB and TNB have been linked to poor survival and an unfavorable response to immunotherapy (23). Further, we randomly divided TCGA samples into two subgroups with a 2:1 ratio by 100 times, and univariate cox regressions in both subgroups demonstrated a significant association of m6A-LRS with overall survival, suggesting a robust predictive value of m6A-LRS (**Supplementary Table S3**). Taken together, our data suggested the clinical value of m6A-LRS in predicting the prognosis and immune response in bladder cancer.

### Validation of m6A-LRS and Association With Immunotherapy Response in IMvigor210 Cohort

**Figure 3:**
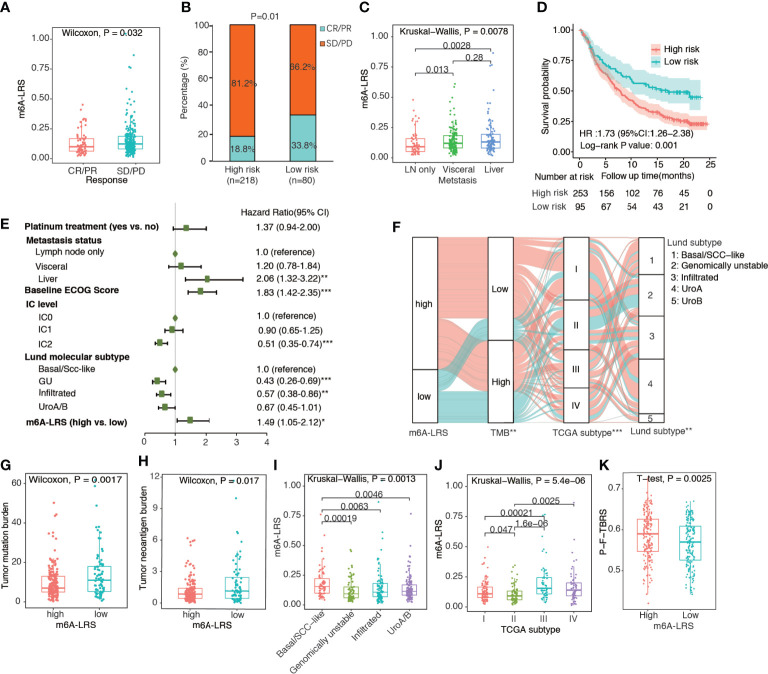
Validation of the m6A-related lncRNA risk score in the IMvigor210 cohort. **(A)** Distribution of m6A-related lncRNA risk score (m6A-LRS) by response status. CR, complete response; PR, partial response; SD, stable disease; PD, progressive disease. **(B)** Comparison of response rates between the high- and low-risk subgroups. **(C)** Distribution of m6A-LRS by metastasis status; **(D)** Kaplan–Meier curves reveal worse overall survival in the high-risk than the low-risk subgroup. **(E)** Forest plot of the multivariate Cox regression results showing the independent predictive value of m6A-LRS after controlling for other confounders. Only variables with a p-value<0.05 in the univariate Cox regression were included in the multivariate model. IC: percentage of PD-L1-positive immune cells (IC) stained under immunohistochemically staining: IC0 (<1%), IC1 (≥1% but <5%), and IC2/3 (≥5%). *P < 0.05, **P < 0.01, ***P < 0.001. **(F)** Alluvial diagram showing the changes in m6A-LRS, FMone tumor mutation burden (TMB), TCGA molecular subtype, and Lund molecular subtype in the IMvigor210 dataset. *P < 0.05, **P < 0.01, ***P < 0.001 for chi-square test between m6A-LRS subgroups and the corresponding variables. **(G, H)** Comparison of TMB and tumor neoantigen burden (TNB) between the high- and low-m6A-LRS subgroups. **(I, J)** Distribution of m6A-LRS based on TCGA molecular subtypes and Lund molecular subtypes. **(K)** Comparison of pan-fibroblast-TGF-β response signature score (pan-F-TBRS) between the high- and low-risk subgroups.

To validate the prognostic value of m6A-LRS, we calculated the corresponding risk score for each patient in the IMvigor210 cohort *via* the same formula. Patients were assigned into high- and low-risk subgroups based on the optimal m6A-LRS cut-off value as determined using the “sur-cut” R function. As shown in **Figures 3A, B** and **Supplementary Table S2**, we observed a significantly higher m6A-LRS in patients responding to anti-PD-L1 treatment. The response (complete response or partial response) rate was almost doubled in the low–risk subgroup compared with that in the high-risk subgroup (33.8% vs. 18.8%, P-value=0.01). Additionally, m6A-LRS was significantly higher in patients with visceral and liver metastases compared with those with only lymph node metastasis (**Figure 3C**). The Kaplan-Meier plot and multivariable Cox regression analyses demonstrated that m6A-LRS was an independent predictor for poorer overall survival, even after controlling for major confounders (**Figures 3D, E** and **Supplementary Table S4**). Taken together, our results indicated that m6A-LRS could serve as an independent predictor for the prognosis and response to immunotherapy in patients with bladder cancer.

TMB has been widely used as a predictive biomarker for cancer immunotherapy. Accordingly, we observed a significantly lower TMB and TNB values in high-risk tumors, suggesting decreased immunogenicity associated with high m6A-LRS (**Figures 3F–H**). Using the Lund molecular taxonomy, we found that m6A-LRS was the highest in the basal/SCC-like subtype and lowest in the genomically unstable subtype, consistent with the importance of TMB. Additionally, TCGA molecular taxonomy revealed that the luminal II subtype, which was similarly enriched for high TMB tumors, exhibited the lowest m6A-LRS (**Figure 3F, I, J**). We also observed that significantly enhanced pan-F-TBRS was associated with m6A-LRS-high tumors (**Figure 3K**), suggestive of the activation of TGF-β signaling in fibroblasts which restrain antitumor immunity. Taken together, our data suggested that the m6A modifications of lncRNA might contribute in the shaping of TME and the modulation of tumor immune evasion, resulting in attenuated responses to immunotherapy.

### Construction of the m6A-LRS-Based Nomogram

**Figure 4:**
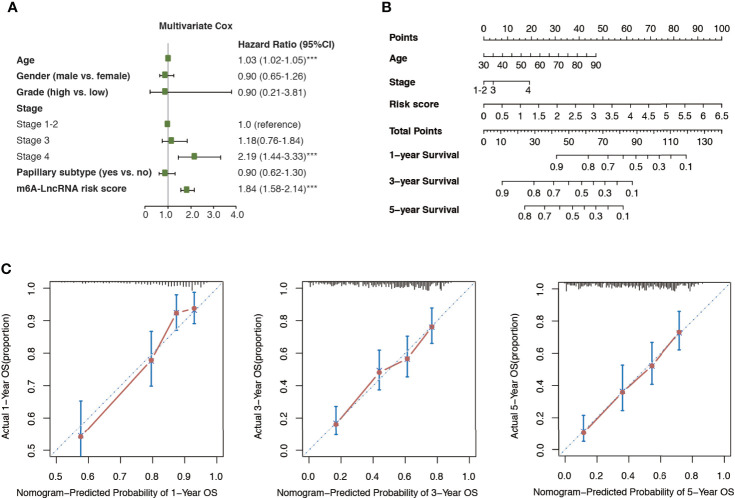
Construction of m6A-LRS nomogram. **(A)** Multivariate Cox regression revealed that the m6A-related lncRNA risk score (m6A-LRS) was an independent prognostic predictor of overall survival in the TCGA dataset. ***P < 0.001. **(B)** Nomogram based on the age, clinical stage, and m6A-LRS. **(C)** Calibration plots of the nomogram for the prediction of overall survival at 1, 3, and 5 years in the TCGA dataset.

Multivariate Cox regression analysis revealed that age, cancer stage, and m6A-LRS were significant independent prognostic factors for overall survival in the TCGA dataset (**Figure 4A**). Therefore, we constructed a nomogram comprised of m6A-LRS, age, and stage (**Figure 4B**). Calibration plots showed that the observed vs. predicted proportion of 1-, 3-, and 5-year overall survival exhibited good concordance in the TCGA dataset (**Figures 4C**). Moreover, a C-index of 0.725 suggested acceptable predictive power. These data suggested that the m6A-LRS-based nomogram might serve as a robust tool for the prediction of survival in patients with bladder cancer.

### Functional Annotation, Gene Set Enrichment Analysis, and Gene Set Variation Analysis

**Figure 5:**
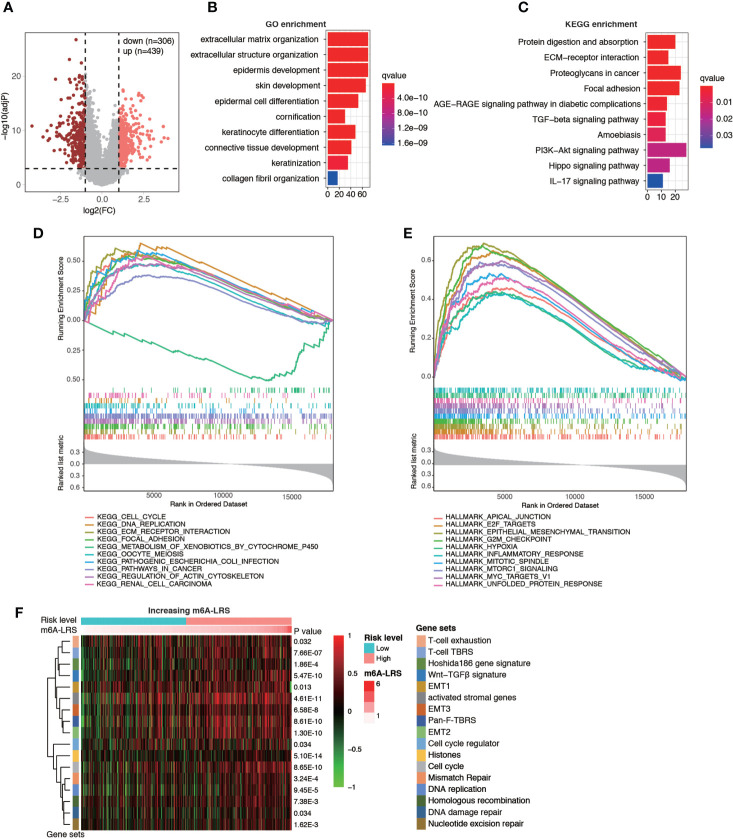
Functional analysis of genes associated with the m6A-related lncRNA risk score. **(A)** Volcano plot showing the distribution of differentially-expressed genes (DEGs) between high- (top 25%) and low-risk (bottom 25%) subgroups. **(B, C)** Top 10 enriched Gene Ontology (GO) and Kyoto Encyclopedia of Genes and Genomes (KEGG) pathways of DEGs. **(D, E)** Gene set enrichment analysis (GSEA) of KEGG pathways and tumor hallmarks associated with m6A-LRS. **(F)** Gene set variation analysis showing the activation states of biological and immune signatures in the high- and low-risk subgroups. A heatmap was used to visualize signatures.

Next, enrichment analysis was performed to investigate the potential biological processes affecting m6A-LRS. Using the TCGA cohort, 745 DEGs were identified between the high- and low-risk subgroups (**Figure 5A**). These genes were primarily enriched in stromal and carcinogenic activation pathways, such as extracellular matrix organization (GO term), extracellular structure organization (GO term), collagen fibril organization (GO term), ECM-receptor interaction (KEGG pathway), focal adhesion (KEGG pathway), and TGF-beta signaling pathway (KEGG pathway) (**Figures 5B, C** and **Supplementary Tables S5** **,** **S6**). In line with these findings, subsequent GSEA on KEGG pathway analysis revealed that tumors with high m6A-LRS exhibited an obvious enrichment of pathways involved in cell cycle, DNA replication, and ECM receptor interaction, etc. In contrast, the “metabolism of xenobiotics by cytochrome P450” KEGG pathway was suppressed in patients with a high m6A-LRS (**Figure 5D** and **Supplementary Table S7**). In addition, tumor hallmarks, such as apical junction, EMT, G2M checkpoint, and hypoxia, were also significantly enriched in tumors with a high m6A-LRS (**Figure 5E** and **Supplementary Table S8**). Consistent with the above findings, our GSVA results also demonstrated that tumors with a high m6A-LRS were associated with the activation of canonical carcinogenic signatures, such as EMT, cell cycle, and DNA replication, as well as immune suppression-associated signatures, such as T-cell exhaustion, T-cell TRBS and pan-F-TBRS (**Figure 5F**), providing potential mechanisms underlying the poorer survival associated with high m6A-LRS.

### Tumor Microenvironment Cell Infiltration

**Figure 6:**
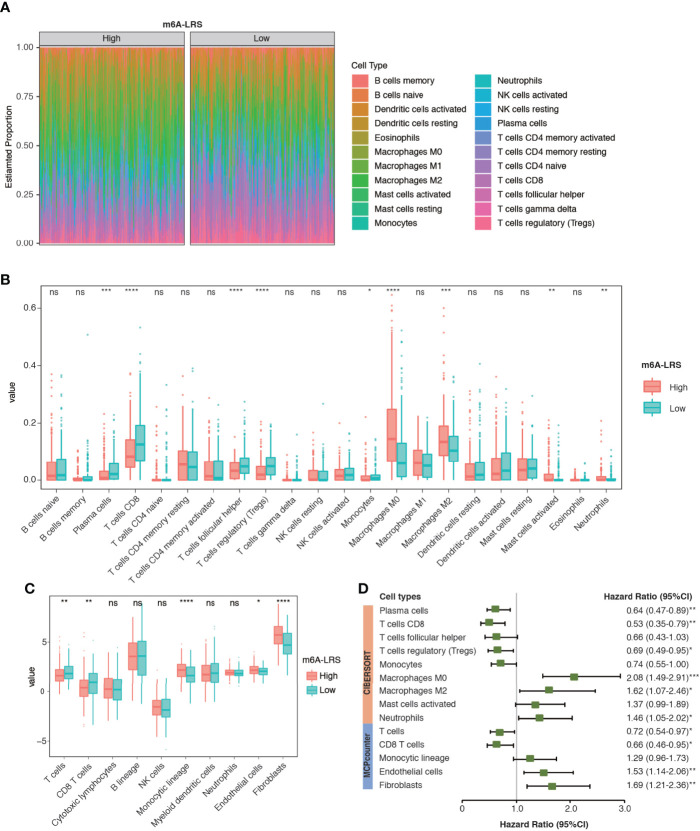
Characteristics of TME cell infiltration in high- and low m6A-LRS subgroups in TCGA. **(A)** The TME infiltrating cell profile in the high- and low-risk subgroups. Relative abundances of 22 cell types were calculated using the CIBERSORT algorithm. **(B)** Comparison of the abundances of infiltrating cells in high- and low-risk subgroups. The upper and lower ends of the boxes represent the interquartile range of values. The lines in the boxes represent median values, and dots show the outliers. **(C)** Comparison of TME stromal and immune cell abundances estimated by “MCPcounter” in the high- and low-risk subgroups. **(D)** Univariate Cox regression of the differential cell types for overall survival. The optimal cut-off value used for grouping was determined using the “sur_cutpoint” function. m6A-LRS: m6A-related lncRNA risk score. *P < 0.05; **P < 0.01; ***P < 0.001, ****P < 0.0001. ns, non-significant.

TME cell infiltration is critical for carcinogenesis and therapeutic response of tumors. To further understand whether the m6A modification of lncRNAs plays important roles in shaping the TME, we investigated the abundance of different immune cell types in the high-risk compared with those in the low-risk subgroups in the TCGA dataset. Analyses of immune cell types using CIBERSORT revealed distinct TME cell-infiltrating characteristics between tumors with high- and low-m6A-LRS. In particular, we found that tumors with higher m6A-LRA were remarkably abundant in macrophages (M0/M2), activated mast cells and neutrophils, but short in plasma cells, CD8 T-cells, follicular helper T-cells, regulatory T-cells (Treg), and monocytes (**Figures 6A, B**). Similarly, TME cell infiltration estimated by the “MCPcounter” algorithm also demonstrated that higher m6A-LRS was associated with reduced infiltration of T-cells/CD8+ T-cells, but enriched infiltration of monocytic lineage cells, endothelial cells, and fibroblasts (**Figure 6C**). Subsequent analysis revealed a matching survival advantage/disadvantage pattern of the significant differential immune cell types. We noticed that macrophages (M0/M2), neutrophils, endothelial cells, and fibroblasts, which were remarkably enriched in tumors with a high m6A-LRS, were significantly associated with poorer survival, whereas T-cells, CD8+ T-cells, Treg, and plasma cells that were significantly enriched in low m6A-LRS tumors exhibited significant survival benefit (**Figure 6D**). Taken together, our results suggested an indispensable role of the m6A methylation of lncRNAs in shaping the TME and inhibiting the antitumor immune response.

### Construction of the ceRNA Network and Functional Enrichment

**Figure 7:**
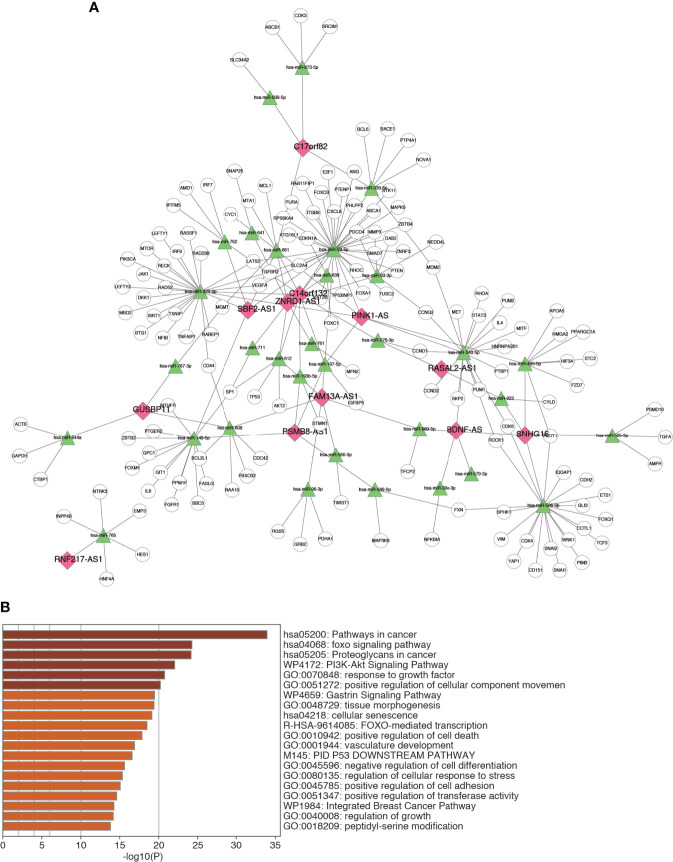
Construction of ceRNA network. **(A)** The ceRNA network of the 12 m6A-related lncRNAs and their target miRNAs and mRNAs. **(B)** Top 20 enriched terms across the 148 target mRNAs.

A major regulatory mechanism of lncRNAs is to function as ceRNAs, thereby modulating the expression of mRNAs. To further explore the potential mechanism affecting m6A-LRS, we constructed a ceRNA network based on the m6A-related lncRNAs. A total of 32 miRNAs were identified based on the 12 lncRNAs according to the analysis of miRanda. Consecutively, using the miRTarBase database we identified 148 target mRNAs based on these 32 miRNAs. Ultimately, 12 lncRNAs, 32 miRNAs, and 148 mRNAs were used to construct the ceRNA network *via* Cytoscape (**Figure 7A** and **Supplementary Table S9**). The 148 target mRNAs were subjected to functional enrichment analysis using the “Metascape” online tool. As shown in **Figure 7B**, these genes were mostly enriched in the “pathway in cancer”, “foxo signaling pathway”, “proteoglycans in cancer”, “PI3K-AKT signaling pathway”, and “response to growth factor”, among others (**Figure 7B**). These data might facilitate the exploration of the functions and regulatory mechanisms of these m6A-related lncRNAs in bladder cancer.

### M6A Modification Site Prediction and MeRIP Validation

**Figure 8:**
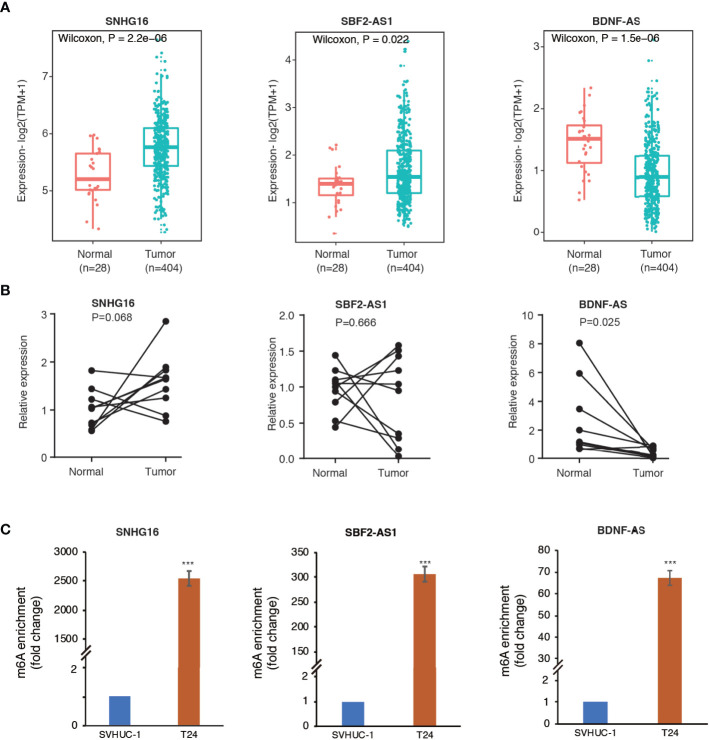
Methylated RNA immunoprecipitation (MeRIP) confirmed m6A enrichment in three selected lncRNAs. **(A)** The differential expression of SNHG16, SBF2-AS1, and BDNF-AS in bladder cancer samples compared to adjacent normal controls in TCGA and GTEx RNA-seq data. **(B)** The expression levels of the three lncRNAs in ten pairs of clinical samples. **(C)** The bar plots indicate relative m6A enrichment in T24 cells in comparison to SVHUC1 cells by MeRIP-qPCR assay. ***P < 0.001.

The SRAMP online tool revealed high confidence of m6A modification on the 12 identified m6A-related lncRNAs. The predictive sites of m6A modification on these lncRNAs are shown in **Supplementary Figure S3**. Then, we selected three lncRNAs (SNHG16, SBF2-AS1, and BDNF-AS) with high confidence of m6A-modification for further validation. As shown in **Figures 8A, B**, analysis using TCGA and GTEx RNA-seq data showed a significant upregulation of SNHG16 and SBF2-AS1 and down-regulation of BDNF-AS in bladder cancer samples compared to normal control samples. In consistence with the RNA-seq data, RT-qPCR analysis on clinical samples also revealed enhanced expression of SNHG16 and decreased expression of BDNF-AS in tumors compared to adjacent normal controls, although the significance of SNHG16 was only marginal (P=0.068), probably due to small sample size. However, no significant difference in the expression level of SBF2-AS1 was observed (P=0.666). Furthermore, MeRIP-qPCR analysis revealed a robust m6A enrichment on these three lncRNAs in T24 bladder cancer cells compared to SVHUC-1 uroepithelial cells (**Figure 8C**), suggesting an enhanced m6A modification in bladder cancer.

## Discussion

Accumulating studies have demonstrated the indispensable roles of the m6A modifications and lncRNAs in tumorigenesis and innate immunity. However, the clinical relevance of the m6A modification of lncRNAs in bladder cancer remains unclear. In this study, we identified m6A-related lncRNAs and constructed a 12 m6A-related lncRNA signature that was closely associated with clinicopathological features, including tumor stage, histological grade, molecular subtype, tumor mutation load, and tumor neoantigen load. Even after controlling for major confounders, the m6A-LRS remained independently predictive of the prognosis and response to immunotherapy, highlighting its potential as a guiding biomarker for individualized therapy. Further, tumors with high- and low-m6A-LRS exhibited distinct TME cell-infiltration characteristics, with the immunosuppressive phenotype being more common in the m6A-LRS-high tumors. Moreover, the MeRIP-qPCR assay confirmed m6A enrichment on three selected lncRNAs in T24 bladder cancer cells in comparison to SVHUC-1 normal uroepithelial cells. Our study might thus contribute to the understanding of m6A modifications in cancer progression and antitumor immune response, providing important implications for more effective immunotherapy strategies.

The m6A modification of lncRNA has been suggested as critical for cancer progression in several types of cancer (34, 35). For instance, the m6A modification of the NEAT1 lncRNA promoted the bone metastasis of prostate cancer by facilitating the CYCLINL1-CDK19 interaction, which was required for the ser2 phosphorylation of Pol II (34). Likewise, METTL3- and METTL14-mediated m6A modification of LncAROD enhanced its stability and promoted ternary complex formation with HSPA1A and YBX1, driving progression in head and neck squamous cell carcinoma (35). Several of the m6A-related lncRNAs included in our prognostic model have previously been reported to be important in cancer progression (36–38). For instance, the SBF2-AS lncRNA, which was associated with poor prognosis in the current study, has been reported to promote malignant progression and chemotherapy resistance in lung cancer (38). In addition, SNHG16 has be suggested to promote EMT by upregulating ITGA6 through a miR-488 inhibition in osteosarcoma (39). Interestingly, our previous study revealed that METTL3 promoted the progression of bladder cancer by enhancing IGTA6 expression *via* m6A modification (33). Taken together, our study identified the prognostic lncRNAs that m6A regulators might target, thereby providing novel insight into their potential roles in the progression of bladder cancer.

In recent years, molecular characteristics have been recognized as critical determinants in the prognosis and treatment responses of bladder cancer (26, 40). In this study, we observed the highest m6A-LRS value in neuronal subtype and lowest value in luminal-papillary subtype in TCGA dataset. In support of our findings, a previous study reported the worst survival in the neuronal subtype and best survival in the luminal-papillary subtype (26). Additionally, using the Lund molecular classifier, we observed the lowest m6A-LRS value associated with genomically unstable subtype, in agreement with a previous study showing the best response to PD-L1 blockade treatment in genomically unstable bladder cancers (23). Taken together, these findings suggested the prognostic significance of m6A-LRS, and highlighted the cross-talk between m6A modification of lncRNAs and molecular events which might contribute to the tumorigenesis of bladder cancer.

Our data revealed a marked enrichment of tumorigenesis-related pathways and hallmarks, such as the cell cycle, DNA replication, ECM receptor interaction, focal adhesion, and EMT, in tumors with a high m6A-LRS, hinting the underlying regulatory mechanisms of the m6A methylation of lncRNAs in the progression of bladder cancer. In addition, TGF-β-related signatures, including the T-cell TBRS, Wnt-TGF-β signature, and the pan-F-TRBS signature, were also enriched in high m6A-LRS tumors. Previous studies have suggested that the activation of TGF-β-related pathways attenuated T-cell infiltration into tumors, compromising tumor killing effects and the immunotherapy response (41, 42). Consistently, we observed here an immunosuppressive phenotype associated with T-cell exhaustion, stromal cell activation, and a lower neoantigen load in high m6A-LRS tumors. The activation of stromal cells has been extensively demonstrated as related to immune evasion, therapeutic resistance, and malignant progression of cancer (43, 44), while the decreased neoantigen load might reflect impaired immunogenicity and a consequently reduced sensitivity to immune checkpoint inhibitors (45, 46). The above findings suggested the important role of the m6A methylation of lncRNAs in immune regulation and treatment resistance, both of which involve the complex interactions of multiple pathways and components.

The TME, which harbors multiple immune and stromal cell types, is a key determinant of tumor progression and antitumor immunity (27, 47). Relationships between m6A regulation and TME immune cell infiltration have been previously demonstrated (24, 48). For instance, Zhang et al. reported three m6A modification patterns with distinct TME infiltration characteristics in gastric cancer, highly consistent with the three major tumor immune phenotypes, including the immune-excluded, immune-inflamed, and immune-desert phenotypes (24). In our study, we also observed distinct TME cell-infiltrating characteristics associated with m6A-LRS. More specifically, high m6A-LRS tumors exhibited immunosuppressive features, such as decreased infiltration of T-cells/CD8+ T-cells and enhanced infiltration of fibroblasts and macrophages (M0/M2). In support of our findings, CD8+ T-cells have been well recognized as major drivers of antitumor immunity (49). Interestingly, a previous study reported that the durable neoantigen-specific immunity was regulated by m6A methylation through the m6A-binding protein YTHDF1, and loss of YTHDF1 in classical dendritic cells resulted in an enhanced antigen-specific CD8+T-cell antitumor response (13). Moreover, in agreement with our findings, tumor-associated macrophages are critical immunosuppressive cells driving tumorigenesis and metastasis (50, 51). Uncommitted macrophages (M0) differentiate into either of two main phenotypes, designated M1 and M2, upon activation. While M1 macrophages have proinflammatory and antitumor activity, M2 macrophages have been widely regarded as important accomplices in tumor progression and have been associated with poor outcomes (52). In addition, cancer-associated fibroblasts, the most prominent stromal cell type within the TME, facilitate an immunosuppressive and growth-promoting microenvironment surrounding tumors, functioning as key determinants of antitumor immunity (53, 54). Taken together, our study suggested the important roles of the m6A methylation of lncRNA in shaping the TME for immune evasion, providing novel insights for effective cancer immunotherapy.

The major limitation of the current study is insufficiency in experimental validation. Although we performed MeRIP-qPCR assay to confirm enhanced m6A modification on three identified m6A-related lncRNAs in bladder cancer cells, the other m6A-related lncRNAs remain unexplored. Besides, the mechanisms by which these m6A-related lncRNAS cooperate with each other in shaping the TME and driving immune evasion remain unclear. Despite these limitations, our study systematically identified m6A-related lncRNAs and provided m6A-LRS as an independent prognostic biomarker for predicting survival and response to immunotherapy. More importantly, we revealed distinct TME cell-infiltrating characteristics associated with m6A-LRS. Our findings provide novel insights for the future development of novel therapeutic strategies. Further experimental studies are warranted to confirm the regulation of these lncRNAs through m6A modification and delineate the corresponding mechanisms in the progression of bladder cancer and immune evasion. Studies with larger sample sizes are also needed to confirm the prognostic value of m6A-LRS.

In conclusion, in this study, we developed and validated an m6A-related lncRNA score with robust prognostic value and the ability to predict response to immunotherapy in patients with bladder cancer. Our study enhanced the understanding of the m6A methylation in TME cell infiltration and immune evasion, providing novel insights for guiding more effective immunotherapy strategies.

## Data Availability Statement

Publicly available datasets were analyzed in this study. This data can be found here: https://xena.ucsc.edu/welcome-to-ucsc-xena; http://research-pub.gene.com/IMvigor210CoreBiologies/.

## Ethics Statement

The studies involving human participants were reviewed and approved by Medical Ethics Committee of The Sixth Affiliated Hospital of Guangzhou Medical University. The patients provided their written informed consent to participate in this study.

## Author Contributions

YZ, BZ, MH, WJ, and JZ conceived the study and its design. YZ, BZ and MH were involved in the data analyses, wrote, reviewed, and edited the manuscript. YC and XY contributed to data analysis and reviewed the manuscript. CJ contributed to the discussion and reviewed the manuscript. WJ and JZ contributed to the discussion, and reviewed, edited and finalized the manuscript. All the authors read and approved the final manuscript. All authors contributed to the article and approved the submitted version.

## Funding

This work was supported by the grants from National Natural Science Foundation of China (No. 81802551, No. 81900688 No. 82073047, No. 81772699, No. 91939109 and No. 81800590), Chinese Postdoctoral Science Foundation (2020M672593), the Medical research Foundation of Guangdong Province (B2020011 and A2019473), the Project of Guangdong Foundation Construction for Science and Technology (2019B030316024), the Guangdong Provincial Joint Fund Youth Project (2020A1515110117), the Natural Science Foundation of Guangdong Province (2016A030307033 and 2019A1515011107), and the Medical Research Foundation of Qingyuan People’s Hospital (No. 20190206, 20190205).

## Conflict of Interest

The authors declare that the research was conducted in the absence of any commercial or financial relationships that could be construed as a potential conflict of interest.

## Publisher’s Note

All claims expressed in this article are solely those of the authors and do not necessarily represent those of their affiliated organizations, or those of the publisher, the editors and the reviewers. Any product that may be evaluated in this article, or claim that may be made by its manufacturer, is not guaranteed or endorsed by the publisher.

## Supplementary Material

Supplementary Figure 1 The mRNA expression levels of 23 m6A regulators in bladder cancer and normal control samples in TCGA and GTEx datasets. Wilcoxon test was used for comparison. **P*<0.05, ***P*<0.01, ****P*<0.001.

Supplementary Figure 2 Kaplan–Meier curves of the 12 m6A-related lncRNAs for overall survival. Best cut-off value used for grouping was determined by the “sur_cutpoint” function.

Supplementary Figure 3 The SRAMP prediction of m6A methylation site on the 12 m6A-related lncRNAs.

## Abbreviations

m6A, N6-methyladenosine; TCGA, The Cancer Genome Atlas; LASSO, least absolute shrinkage and selection operator; lncRNA, long non-coding RNA; m6A-LRS, prognostic score based on12 m6A-related lncRNAs; TPM, transcripts per kilobase million; TNB, tumor neoantigen burden; TMB, tumor mutation burden; GO, Gene Ontology; KEGG, Kyoto Encyclopedia of Genes and Genomes; TME, tumor microenvironment; GSEA, gene set enrichment analysis; GSVA, gene set variation analysis; ceRNA, competing endogenous RNA; EMT, epithelial-to-mesenchymal transition. qPCR: Quantitative real-time PCR (qPCR); MeRIP, methylated RNA immunoprecipitation.
