# Supplementary figures and images for: N6-Methylandenosine-Related lncRNAs Predict Prognosis and Immunotherapy Response in Bladder Cancer
Source: Front Oncol. 2021 Aug 11;11:710767. doi: 10.3389/fonc.2021.710767 (PMC8387102; doi:10.3389/fonc.2021.710767)

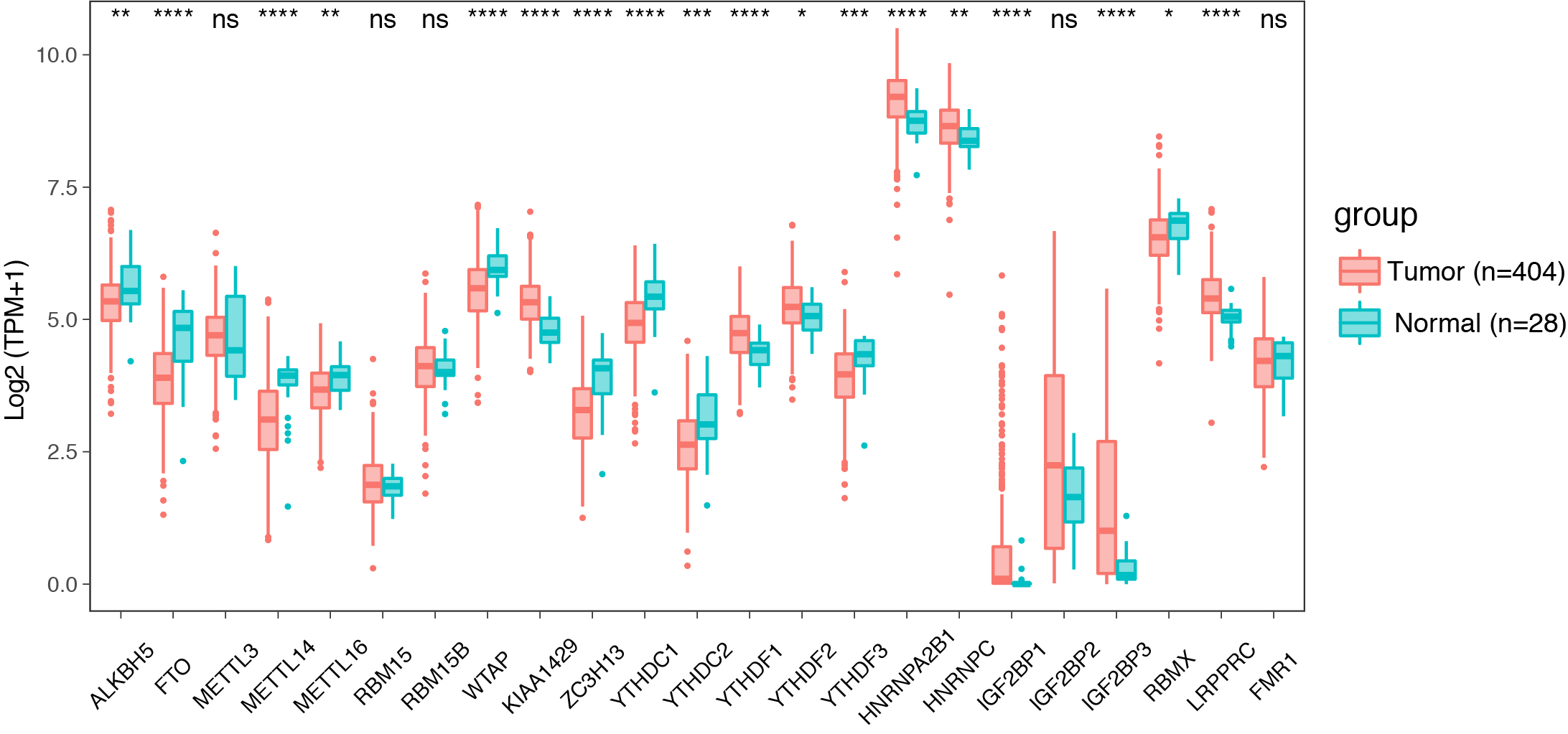

Supplement: Supplementary Figure 1 — The mRNA expression levels of 23 m6A regulators in bladder cancer and normal control samples in TCGA and GTEx datasets. Wilcoxon test was used for comparison. *P<0.05, **P<0.01, ***P<0.001. [file Image_1.jpeg]

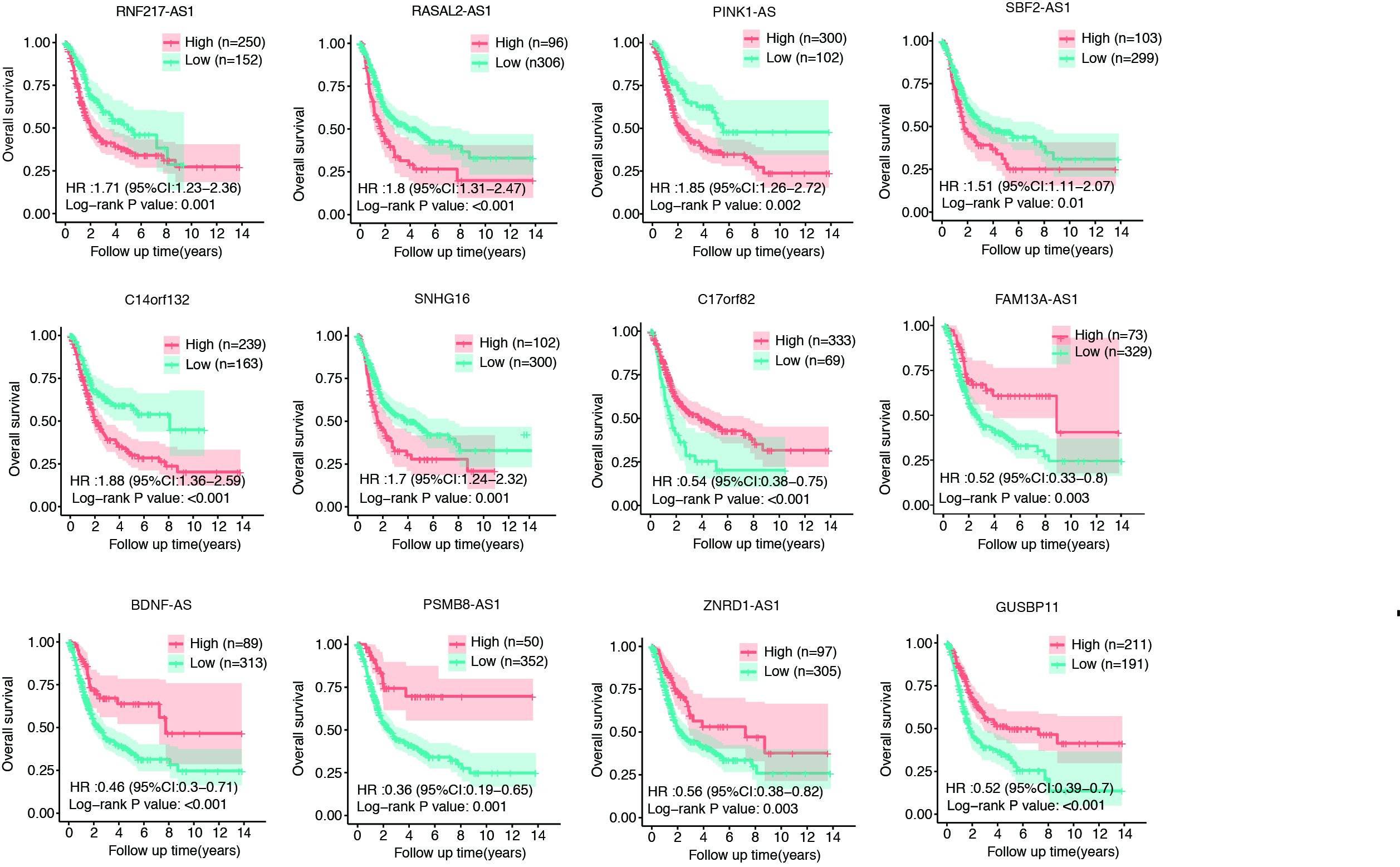

Supplement: Supplementary Figure 2 — Kaplan–Meier curves of the 12 m6A-related lncRNAs for overall survival. Best cut-off value used for grouping was determined by the “sur_cutpoint” function. [file Image_2.jpeg]

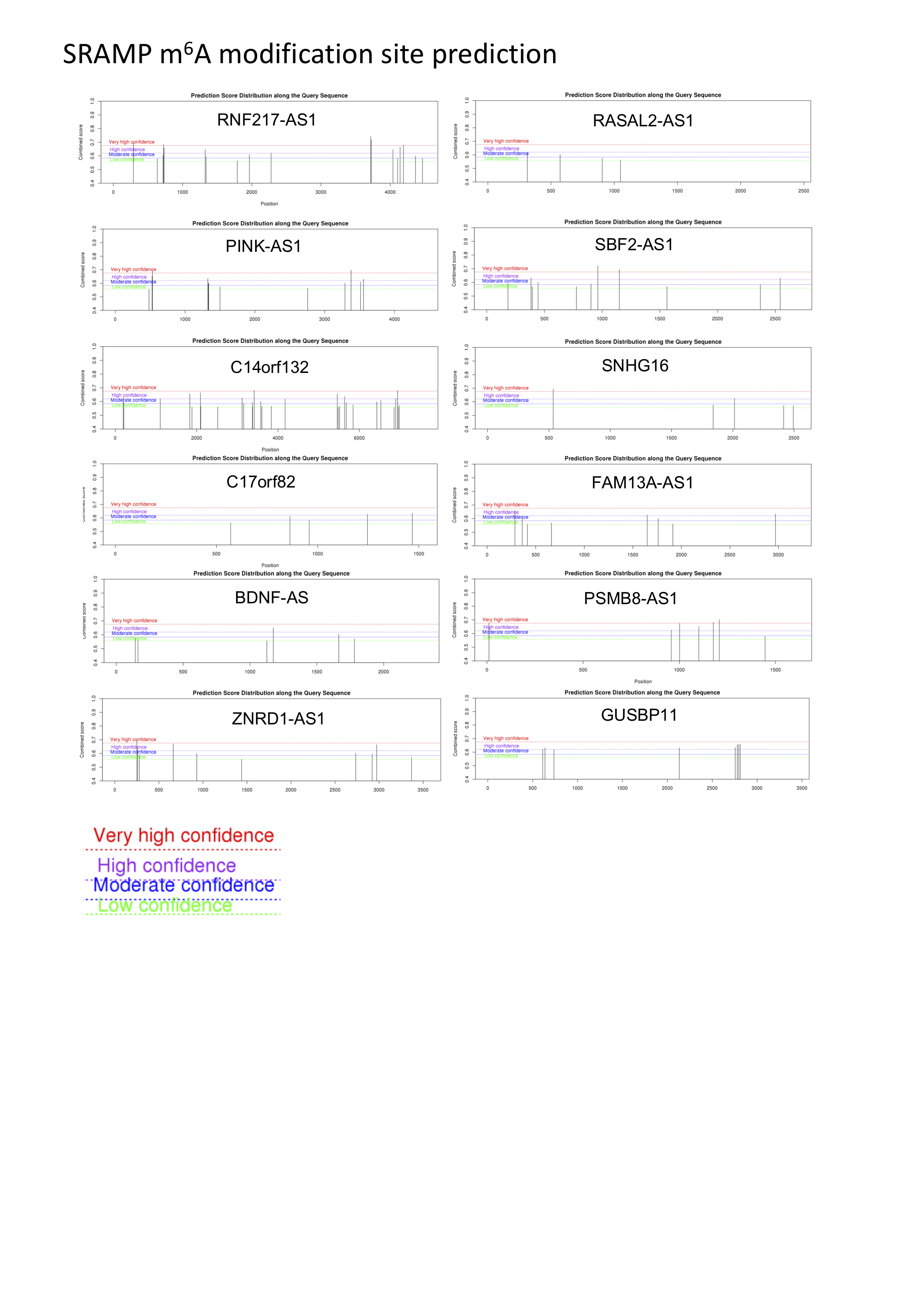

Supplement: Supplementary Figure 3 — The SRAMP prediction of m6A methylation site on the 12 m6A-related lncRNAs. [file Image_3.jpeg]
